# Expression pattern of p53-binding protein 1 as a new molecular indicator of genomic instability in bladder urothelial carcinoma

**DOI:** 10.1038/s41598-018-33761-9

**Published:** 2018-10-19

**Authors:** Katsuya Matsuda, Tatsuhiko Kawasaki, Yuko Akazawa, Yuhmi Hasegawa, Hisayoshi Kondo, Keiji Suzuki, Masachika Iseki, Masahiro Nakashima

**Affiliations:** 1 Department of Tumor and Diagnostic Pathology, Atomic Bomb Disease Institute, Nagasaki University, Nagasaki, Japan; 2 Department of Pathology, Sasebo Kyosai Hospital, Nagasaki, Japan; 3 Department of Gastroenterology and Hepatology, Nagasaki University Hospital, Nagasaki, Japan; 4 Medical Student Research Program, Nagasaki University School of Medicine, Nagasaki, Japan; 5 Biostatistics Section, Division of Scientific Data Registry, Atomic Bomb Disease Institute, Nagasaki University, Nagasaki, Japan; 6 Department of Radiation Medical Sciences, Atomic Bomb Disease Institute, Nagasaki University, Nagasaki, Japan

## Abstract

Copy number alterations and loss of heterozygosity are associated with increasing tumor grade and bladder cancer stage. Our previous study suggested that co-expression of Ki-67 and p53-binding protein 1 (53BP1) could provide an indicator of an abnormal DNA damage response (DDR) pathway. The present study investigated 53BP1 expression as a novel molecular marker in urothelial carcinoma (UC) using bladder tissues with in total of 40 cases including a normal urothelium, urothelial papilloma, low-grade UC, or high-grade UC. Double-label immunofluorescence was used to analyze 53BP1 and Ki-67 expression. This was compared with the level of chromosomal instability and with the expression of other DDR molecules catalytic subunit. This study identified clear differences in the 53BP1 expression patterns in urothelial carcinogenesis, and their close association with genomic instability. 53BP1 abnormal immunoreactivity, particularly with co-localization of Ki-67, was restricted to malignant tissues. Our analyses indicated that a cut-off of >4% of nuclei with 53BP1 abnormal expression plus Ki-67 immunoreactivity distinguished high-grade UC from low-grade UC with 80.0% sensitivity and 100% specificity. We therefore propose that double immunofluorescent analysis of 53BP1 and Ki-67 expression could provide a useful tool to estimate the chromosomal instability and malignant potential of urothelial tumors.

## Introduction

Urothelial carcinoma (UC) is a malignant neoplasm derived from the urothelial cells that accounts for more than 90% of bladder tumors. UC can be classified histologically as low-grade (LG) or high-grade (HG)^1,2^ with implications for the clinical behavior of the tumor^3–5^. However, this histological classification is not always straightforward and may be affected by inter-observer variability. Therefore, ancillary immunohistochemical or molecular studies are required to enhance reproducibility and improve the correlation with clinical outcome^6^.

The risk of bladder cancer is increased by smoking, exposure to occupational carcinogenic materials such as the aromatic amines found in chemical dyes, local chronic inflammation, and iatrogenic factors such as treatments involving anticancer agents and radiotherapy^7–14^. Exposure of the bladder mucosa to various carcinogenic compounds excreted in urine may induce DNA damage in urothelial cells; this leads to genomic instability (GIN), a key carcinogenic mechanism^15,16^. Recent efforts involving next-generation sequencing have demonstrated complex mutational landscapes in urothelial tumors, with >300 mutations, >200 copy number alterations and >20 rearrangements per tumor^15,17,18^. These data suggested that increases or decreases in copy number and loss of heterozygosity, which are indicators of GIN, were associated with increased tumor grade and bladder cancer stage^19^.

GIN is induced by functional abnormalities of the DNA damage response (DDR) pathway and plays a significant role in tumorigenesis^20,21^. The p53-binding protein 1 (53BP1) belongs to a family of evolutionarily conserved DDR proteins with BRCA1 C-terminus domains^22,23^. Since 53BP1 localizes at sites of DNA double-strand breaks and rapidly forms nuclear foci to activate downstream effectors such as TP53 tumor suppressor molecules^24–29^, the presence of 53BP1 nuclear foci can be used as an indicator of double-strand break sites and of DDR activation. Our previous study of uterine cervical epithelial neoplasms demonstrated that the pattern of 53BP1 immunoreactivity could be classified into four types: (1) stable (no staining); (2) low DDR (one or two discrete nuclear foci); (3) high DDR (three or more discrete nuclear foci); and (4) large foci (LF), defined as discrete nuclear foci of ≥1.0 μm^30^. This study revealed that types 3 and 4 (high DDR and large 53BP1 foci) were closely associated with a higher level of cervical carcinogenesis^30^. Interestingly, double staining for 53BP1 and Ki-67 has shown frequent co-localization of 53BP1 nuclear foci and Ki-67 in carcinoma cells, while this was very rare in low squamous intraepithelial lesions of the cervix. Since DDR is normally associated with cell cycle arrest, the presence of 53BP1 nuclear foci and Ki-67, a proliferation marker, can be considered as an indicator of an abnormal DDR pathway, which is subsequently associated with carcinogenesis via GIN^30^.

GIN in UC takes the form of a pronounced chromosomal instability, rather than excessive point mutations^31^. UroVysion^TM^ is a promising test that uses multi-colored FISH and has been approved by the USA FDA; this can be used to grade UCs and estimate the malignant potency of urothelial tumors and, importantly, can differentiate between an inverted papilloma and inverted LGUC^32^. This assay was designed to detect copy number changes for chromosomes 3, 7 and 17, and at the chromosome 9p21 locus. UroVysion^TM^ detects HGUC and carcinoma *in situ* with reliable sensitivity, but shows a lower sensitivity for LGUC^33,34^. Hyper aneuploidy may occur in carcinoma *in situ* and invasive UC^35^, whereas loss at 9p21 has also been associated with non-invasive papillary UC^31,34,35^. The present study clarified the profile of 53BP1 expression in bladder tumor tissues, and its association with the expression of other DDR molecules or chromosomal instability, using the UroVysion^TM^ test. Our results demonstrated that abnormal 53BP1 expression was significantly associated with an abnormal DDR pathway and a high level of copy number alteration in HGUC, suggesting that this could provide a new molecular marker to distinguish HGUC from LGUC and to estimate GIN during urothelial carcinogenesis.

## Results

### Expression of 53BP1 and Ki-67

**Figure 1:**
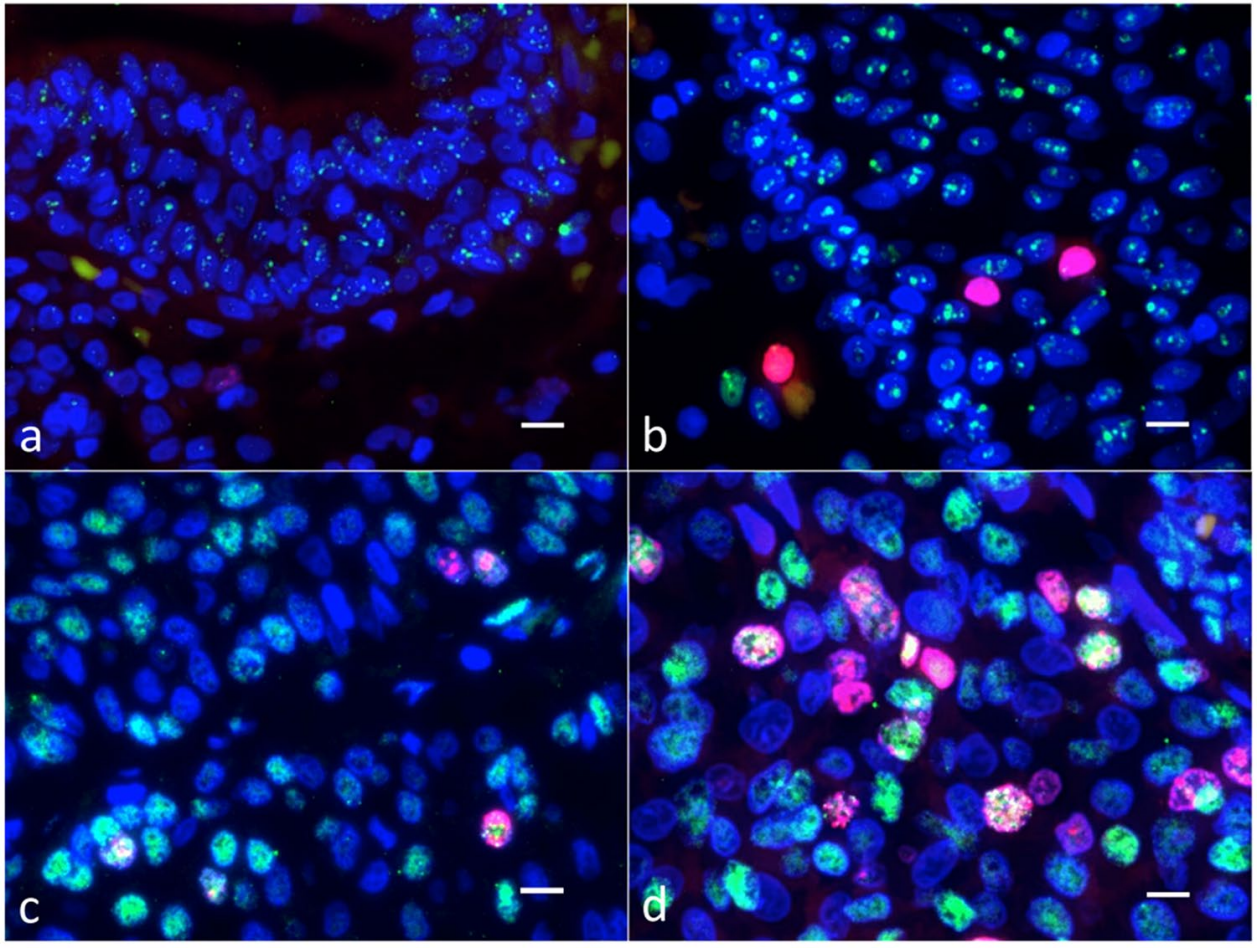
Double-label immunofluorescent studies of p53-binding protein 1 (53BP1) (green) and Ki-67 (red) in urothelial tissues. The normal urothelium (**a**) and urothelial papilloma (**b**) show low and high DNA damage response types of 53BP1 expression, while both the low-grade (**c**) and high-grade (**d**) urothelial carcinomas (UC) show the diffuse expression type. Nuclei showing co-localization of 53BP1 and Ki-67 immunoreactivity were observed in UC tissues (**c**,**d**), and were more frequent in high-grade UC (**d**). The scale bars indicate 10 µm.

The 53BP1 expression types identified in the present study are shown in Table 1, and typical examples are depicted in Fig. 1. Most of the nuclei of the normal urothelium were stable or DDR type, with few diffuse or LF type. The frequency of appearance of diffuse or LF 53BP1 expression patterns in all cells tends to increase significantly with increasing nuclear atypism of UC (p < 0.0001, Jonckheere-Terpstra test). Thus, UC showed a higher frequency of diffuse LF 53BP1 expression patterns, as compared with normal urothelium and UP.

**Table 1:** Types of p53-binding protein1 (53BP1) expression in urothelial tumors.

			Type of 53BP1 expression in all nuclei (%)					Ki-67-positive nuclei	Type of 53BP1 expression in Ki-67-positive nuclei (%)				
	n	Total nuclei	Stable	Low DDR	High DDR	Diffuse	LF		Stable	Low DDR	High DDR	Diffuse	LF
Normal	10	3612	1606 (44.5%)	1096 (30.3%)	834 (23.1%)	31 (0.9%)	45 (1.3%)	226 (6.3%)	127 (3.5%)	44 (1.2%)	51 (1.4%)	4 (0.1%)	0
UP	10	4876	1544 (31.7%)	1881 (38.6%)	1378 (28.3%)	9 (0.2%)	64 (1.3%)	94 (1.9%)	56 (1.1%)	22 (0.5%)	16 (0.3%)	0	0
LGUC	10	4340	1814 (41.8%)	709 (16.3%)	728 (16.8%)	1049 (24.2%)	40 (0.9%)	295 (6.8%)	190 (4.3%)	32 (0.7%)	29 (0.7%)	40 (0.9%)	4 (0.1%)
HGUC	10	3810	2087 (54.8%)	213 (5.6%)	211 (5.5%)	1146 (30.0%)	153 (4.0%)	1152 (30.2%)	635 (16.7%)	84 (2.2%)	146 (3.8%)	235 (6.2%)	52 (1.4%)
p value			p < 0.0001^†^						p < 0.0001^†^				

^†^Jonckheere-Terpstra test. DDR, DNA damage response; LF, large foci; Normal, normal urothelium; UP, urothelial papilloma; LGUC, low grade urothelial carcinoma; HGUC, high grade urothelial carcinoma.

Analysis of nuclear Ki-67 revealed that co-localization with diffuse or LF types of 53BP1 expression was mostly restricted to UCs, with co-localization rates of 0.1%, 0%, 1.0% and 7.6% observed in normal, UP, LGUC and HGUC tissues, respectively. The statistical analysis revealed that the histological urothelial tumor type was significantly associated with the type of 53BP1 expression (p < 0.0001).

### Association between the 53BP1 expression pattern and chromosomal instability

**Figure 2:**
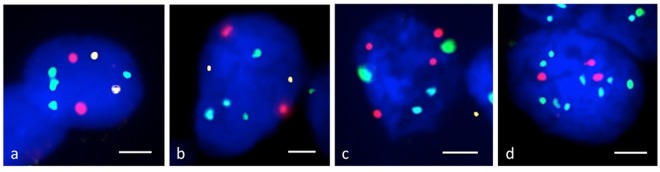
Representative multi-colored FISH images of chromosomes 3 (red), 7 (green), 9p21 (gold) and 17 (aqua) in urothelial tissues. The normal urothelium (**a**) and urothelial papilloma (**b**) showed wild-type expression, whereas urothelial carcinomas (UC) exhibited abnormal genomic signals such as a gain of chromosome 3 and loss of 9p21 in low-grade UC (**c**) and gains of chromosome 3, 7 and 17, with a loss of 9p21, in high-grade UC (**d**). The scale bars indicate 2 µm.

Typical examples of multi-colored FISH analysis are depicted in Fig. 2 and Supplemental Fig. 1. The data relating to the diffuse or LF type of 53BP1 expression, the Ki-67 labeling index, chromosomal abnormalities detected by FISH, and the level of chromosomal instability in each case are presented in Table 2, and the associations between the 53BP1 expression type and the level of chromosomal instability are summarized in Table 3. Our results revealed that multiple chromosomal alterations were found in UC, but not in normal urothelium and UP. Furthermore, 80% of the HGUC samples showed triple or quadruple chromosomal alterations, >30% diffuse or LF 53BP1 expression types, and >3% co-localization of these 53BP1 expression types and Ki-67 (Table 2). Statistical analysis revealed a significant association between the type of 53BP1 expression and the level of chromosomal instability (p < 0.0001; Table 3).

**Table 2:** Types of p53-binding protein 1 (53BP1) expression and chromosomal instability in each case.

Histological type	Case number	Sex	Age	Grade	Prognosis	T.N.M	Type of 53BP1 expression		Ki-67 labelling index	FISH				Level of Chr instability
							Diffuse or LF (%)	Diffuse or LF plus Ki-67 positivity (%)		Chr3	Chr7	9p21	Chr17	
Normal (n = 10)	1	F	81				9.8	0.9	22.4	—	—	Loss	—	1
	2	M	79				9.5	0	0.8	—	—	—	—	0
	3	M	64				0	0	0.3	—	—	—	—	0
	4	M	69				0.7	0	7.8	—	—	—	—	0
	5	M	55				1.2	0	0	—	—	—	—	0
	6	M	86				0	0	1.0	—	—	—	—	0
	7	M	52				5.3	0	9.1	—	—	—	—	0
	8	M	82				0.6	0.2	20.0	—	—	Loss	—	1
	9	M	77				0	0	4.7	—	—	—	—	0
	10	M	82				0.2	0	0	—	—	—	—	0
UP (n = 10)	11	M	67				0.3	0	0.3	—	—	Loss	—	1
	12	M	75				0	0	0	—	—	—	—	0
	13	F	75				0.2	0	3.1	—	—	—	—	0
	14	F	74				0	0	5.8	Gain	—	—	—	1
	15	M	72		Recurrence		0.5	0	4.1	—	—	—	—	0
	16	M	76				2.8	0	1.9	Gain	—	—	—	1
	17	F	59				0.5	0	5.6	Gain	—	—	—	1
	18	M	63				1.1	0	0.7	—	—	—	—	0
	19	F	68				8.7	0	0.2	—	—	—	—	0
	20	M	78				1.3	0	0	—	—	—	—	0
LGUC (n = 10)	21	F	72	1 > 2	Recurrence	TaN0M0	19.5	2.1	20.4	Gain	Gain	Loss	Gain	4
	22	M	63	1		TaN0M0	26.2	0.6	2.7	Gain	—	—	—	1
	23	M	79	1 > 2		TaN0M0	2.6	0	7.6	—	—	Loss	—	1
	24	M	75	1		TaN0M0	11.7	0	6.2	—	—	Loss	—	1
	25	F	87	1	Recurrence	TaN0M0	63.0	1.2	2.8	Gain	—	Loss	—	2
	26	M	66	1		TaN0M0	22.1	0	3.4	—	—	Loss	—	1
	27	M	54	1	Recurrence	TaN0M0	5.9	0	0.3	Gain	—	Loss	—	2
	28	F	82	1		TaN0M0	16.2	0.4	2.1	—	—	Loss	—	1
	29	M	84	2		TaN0M0	22.7	3.0	8.7	Gain	—	Loss	—	2
	30	M	71	2	Recurrence	TaN0M0	45.5	2.5	13.8	—	—	Loss	—	1
HGUC (n = 10)	31	F	75	2		T2bN0M0	56.1	17.5	31.5	Gain	Gain	Loss	Gain	4
	32	F	74	2 > 3	Death	T3N0M0	34.4	3.1	26.3	Gain	—	Loss	—	2
	33	M	76	3		T2aN0M0	36.4	0	4.7	Gain	Gain	Loss	Gain	4
	34	M	74	2 > 3	Death	T2bN0M0	40.2	12.9	31.3	Gain	Gain	Loss	Gain	4
	35	M	70	2 > 3		T1N0M0	31.8	8.1	23.1	Gain	—	Loss	—	2
	36	M	61	2	Recurrence	T2bN0M0	42.4	4.6	17.4	Gain	—	Loss	Gain	3
	37	M	93	3		T2aN0M0	10.9	0.5	19.2	Gain	—	Loss	Gain	3
	38	M	42	2 > 3	Death	T3bN0M0	38.7	7.5	27.9	Gain	—	Loss	Gain	3
	39	M	80	3 > 2	Death	T1N0M0	15.4	7.5	55.0	Gain	Gain	Loss	Gain	4
	40	M	79	3	Death	T1N0M0	26.8	10.5	54.1	Gain	Gain	—	Gain	3

LF, large foci; Normal, normal urothelial epithelium; UP, urothelial papilloma; LGUC, low grade urothelial carcinoma; HGUC, high grade urothelial carcinoma; Chr, chromosome.

**Table 3:** Association between 53BP1 expression type and chromosomal instability in urothelial cells.

Level of chromosomal instability	Number of nuclei	Type of 53BP1 expression				
		Stable	Low DDR	High DDR	Diffuse	LF
0	4966	1890 (38.1%)	1552 (31.3)	1427 (28.7)	10 (0.2%)	87 (1.8%)
1	5897	2293 (38.9%)	1830 (31.0%)	1141 (19.4%)	583 (9.9%)	50 (0.9%)
2	1984	890 (44.9%)	188 (9.5%)	262 (13.2%)	634 (32.0%)	10 (0.5%)
3	2179	1122 (51.5%)	253 (11.6%)	262 (12.0%)	393 (18.0%)	149 (6.8%)
4	1612	856 (53.1%)	76 (4.71)	59 (3.7%)	615 (38.2%)	6 (0.4%)
		p < 0.0001^†^				

^†^Jonckheere-Terpstra test; DDR, DNA damage response; LF, large foci.

### Associations between the 53BP1 expression pattern and DDR-related proteins

**Figure 3:**
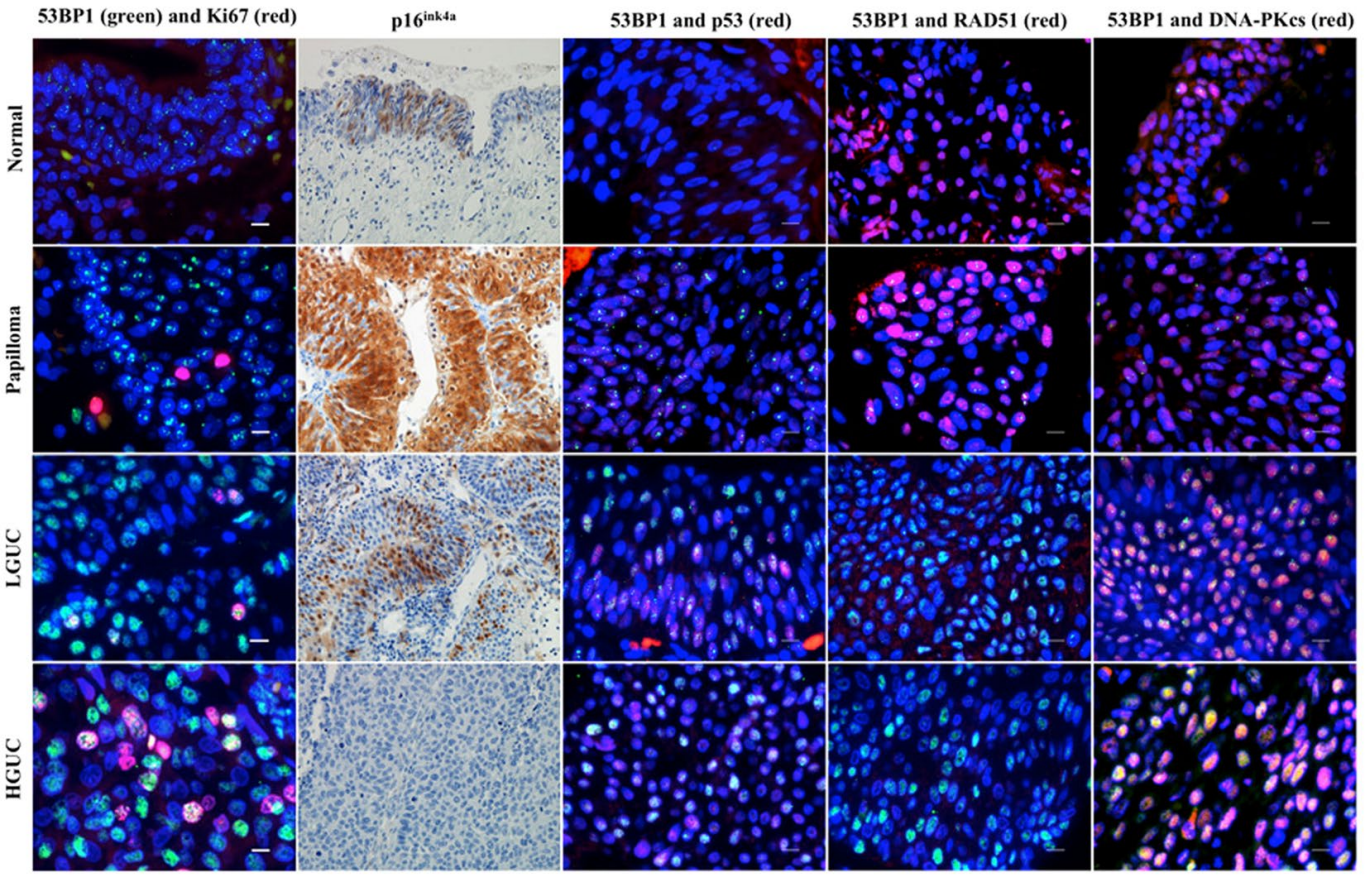
Double-label immunofluorescent and immunohistochemical studies of p53-binding protein 1 (53BP1) expression (green) and Ki-67 (red) in urothelial tissue, along with other DNA damage response (DDR)-related molecules: p16^ink4a^ (brown), p53 (red), RAD51 (red), and DNA-PKcs (red). The scale bars indicate 10 µm.

In uterine cervical tumors, overexpression of p16^ink4a^ inhibits cellular growth in the presence of replication stress^36^. In the present study, p16^ink4a^ immunoreactivity was elevated in UP tissue with the DDR types of 53BP1 expression, and downregulated in LGUC or absent in HGUC with the diffuse or LF 53BP1 expression patterns (Fig. 3). Double-label immunofluorescent analyses revealed a frequent co-localization of the DDR types of 53BP1 expression and γH2AX nuclear foci (Supplemental Fig. 2), and a frequent co-localization of the diffuse or LF types of 53BP1 expression and p53 overexpression in HGUC. In both LGUC and HGUC tissues, a loss of RAD51 immunoreactivity was observed, along with frequent co-localization of the diffuse or LF types of 53BP1 and DNA-PKcs (Fig. 3).

## Discussion

This study identified clear alterations of 53BP1 expression in UC and a close association between these changes and GIN in tumor cells. The number of urothelial cell nuclei with DDR types of 53BP1 expression patterns was greater in non-neoplastic and benign tumors such as UP, while the diffuse or LF types of 53BP1 immunoreactivity, and particularly their co-localization with Ki-67, was restricted to malignant LGUC and HGUC tumors. Double-label immunofluorescent studies also revealed nuclear co-localization of 53BP1 and γ-H2AX in UP, providing further evidence for the presence of DNA double-strand breaks in these cells. Furthermore, our logistic regression analysis indicated that the presence of the diffuse or LF types of 53BP1 expression patterns in >9.8% of the cells could distinguish LGUC from UP with 90.0% sensitivity and 95.0% specificity (area under curve, 0.98; Youden index, 0.85). In addition, the presence of the diffuse or LF types of 53BP1 expression patterns plus Ki-67 in >4% of the nuclei could distinguish HGUC from LGUC with 80.0% sensitivity and 100% specificity (area under curve, 0.87; Youden index, 0.80). These results were consistent with our previous studies showing differences in 53BP1 expression patterns in thyroid, skin and uterine cervical tumors resected from patients^30,37,38^. Taken together, these findings indicate that double immunofluorescent analysis of 53BP1 and Ki-67 expression can provide a useful tool to estimate the level of GIN and the malignant potential of a range of human tumors. The present study indicated that for urothelial tumors, our technique may be useful to detect not only HGUC but also for LGUC with reliable sensitivity. Immunofluorescent analysis is also associated with much lower cost and is technically easier as compared to FISH assay. DNA damage in the urothelium can result from various stimuli including chemicals excreted via the urine and chronic inflammation, and results in GIN^19^. In contrast to thyroid follicular cells^37^ or the cervical epithelium^30^, samples of normal urothelium occasionally showed several nuclear 53BP1 expression foci in the current study. Similarly, our previous study of skin revealed an increased number of nuclear 53BP1 foci in non-neoplastic epidermis at sun-exposed sites, but not at non-exposed sites^38^. In the present study, we found two cases (No. 1 and 8) of normal urothelium with the diffuse and LF types of 53BP1 expression in Ki-67 positive cells. Interestingly, these cases exhibited high Ki-67 labelling indices of around 20% and a loss of 9p21, suggesting a replication stress with GIN, even though the urothelium appeared to be normal. The LDDR and HDDR types of 53BP1 immunoreactivity observed in non-neoplastic bladder urothelium and sun-exposed epidermis tissues may represent minor genotoxic injuries induced by their continuous exposure to external environmental genotoxic factors.

This study demonstrated that the frequent co-localization of diffuse or LF types of 53BP1 expression and Ki-67 provided a marker for cycling cells in UC because this co-localization was rare in normal urothelium and UP. The DDR pathway is normally activated after cell cycle arrest by p53 and it can therefore be hypothesized that co-localization of 53BP1 and Ki-67 in cancer cells indicates a disrupted DDR pathway. To test this hypothesis, we analyzed the associations between 53BP1 expression and other DDR-related molecules such as RAD51, a key molecule for homologous recombination, and DNA-PKcs, a key molecule for non-homologous end joining repair. These analyses revealed that the diffuse and LF types of 53BP1 expression were frequently co-localized with DNA-PKcs expression in UC cells, in association with a loss of RAD51 expression, reduced p16^ink4a^ levels and p53 overexpression; these changes showed tumor progression-related increases in severity, such as UP-LGUC-HGUC. Overexpression of p16 immunoreactivity in UP is considered to represent a response to replication stress^39^, suggesting that the molecular mechanisms involved in regulating the cell cycle are at least partially intact at the benign stage. Loss of 9p21, which is the specific locus coding p16^ink4a^, is frequently seen in LGUC and represents an early critical event during carcinogenesis^16,40–42^. Our immunohistochemical studies confirmed that there was a loss of p16^ink4a^ in UC (including LGUC) and an overexpression of p53 in HGUC. These findings indicated that losses of normal p16^ink4a^ and p53 activities were associated with malignant transformation and progression, respectively, in urothelial carcinogenesis. At the same time, decreased expression of RAD51 and increased expression of DNA-PKcs could lead to errors in non-homologous end joining repair, inducing GIN and the severe chromosomal aberrations observed in HGUC.

In summary, this study demonstrated that UC tissues exhibited diffuse and LF types of 53BP1 expression in cycling cells, suggesting that disruption of DDR allowed subsequent replication of the genomic injury. Furthermore, these abnormal types of 53BP1 expression were closely associated with a high level of chromosomal instability in HGUC, as demonstrated by multi-colored FISH, and with abnormal expression of other DDR molecules, as demonstrated by double immunofluorescent analyses. Thus, we propose that double immunofluorescent analysis of 53BP1 and Ki-67 expression can provide a useful tool to estimate the malignant potential and grade of urothelial tumors. Because this study was retrospectively conducted in a single institute with a small population, a prospective study with a larger cohort should be required to recommend the procedure for routine diagnostic use.

## Materials and Methods

### Bladder tissue

This study investigated bladder lesions resected from a total of 40 individuals: 30 males and 10 females. All samples were resected from patients at the Department of Urology, Sasebo Kyosai Hospital, between 2012 and 2017. The urothelial tumor diagnosis was confirmed by two certificated pathologists (M. I. and M. N.), in accordance with the 4^th^ World Health Organization Classification of Tumours of the Urinary System and Male Genital Organs^2^ Histologically, our transurethral resected bladder tissue samples included 10 cases of normal urothelial epithelium (average age, 71.4 yr), 10 cases of urothelial papilloma (UP; average age, 70.7 yr), LGUC (average age, 73.3 yr) and HGUC (average age, 72.4 yr). The nuclear grade, TNM classification and prognosis of each case are shown in Table 2.

**Figure 4:**
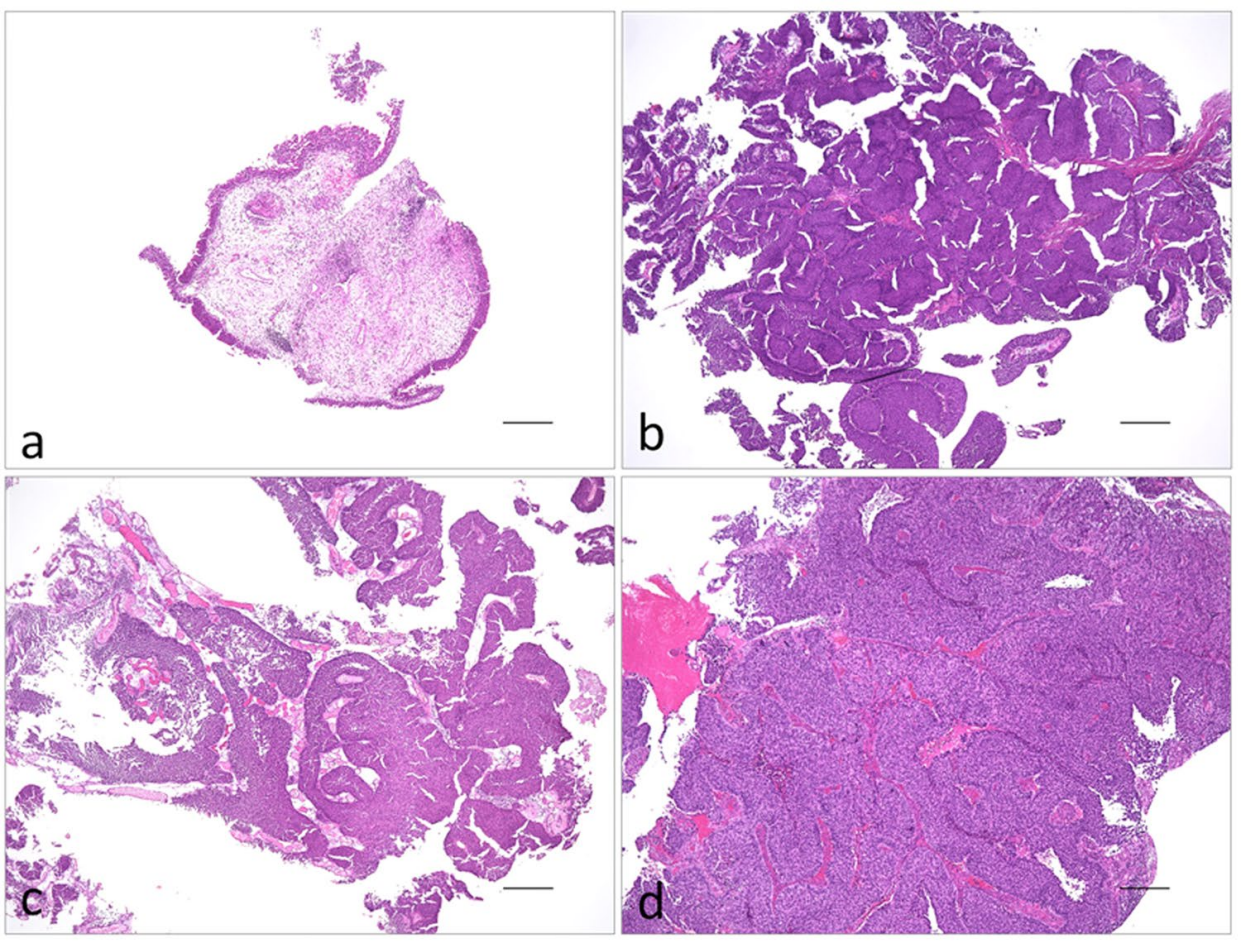
Representative histological images of the tissues used in this study. (**a**) Normal urothelium; (**b**) urothelial papilloma; (**c**) low-grade urothelial carcinoma; (**d**) high-grade urothelial carcinoma. The scale bars indicate 300 µm.

Representative histology images are shown in Fig. 4. All tissues were formalin-fixed, paraffin-embedded, and prepared for immunofluorescence and multi-colored FISH studies (UroVysion^TM^).

This study was conducted retrospectively and in accordance with the tenets of the Declaration of Helsinki. It was approved by the institutional ethical committees for medical research at Nagasaki University (approval date: July 24, 2015; #15062617) and at Sasebo Kyosai Hospital (approval date: Mar 17, 2017; 3^rd^ ethical committee). In accordance with the guidelines of the ethical committee’s official informed consent and disclosure system, detailed information regarding this study is available on our website (http://www-sdc.med.nagasaki-u.ac.jp/pathology/research/index.html). Patients were able to opt out of the study by following the instructions provided on the faculty website.

### Immunofluorescent analyses

**Figure 5:**
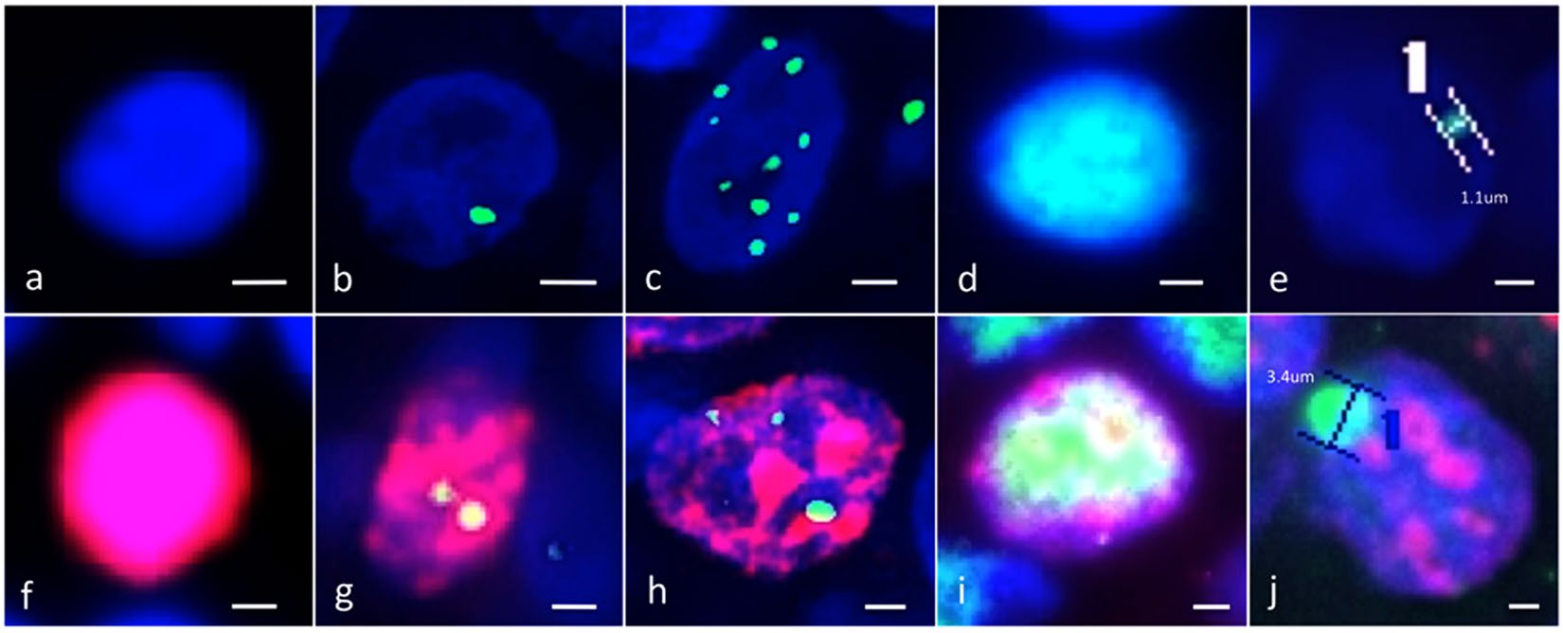
Double immunofluorescent studies identified five types of p53-binding protein 1 expression pattern (green). These were observed in the absence (**a**–**e**) or presence (**f**–**j**) of Ki-67 expression (red). (**a**,**f**) stable type with little nuclear 53BP1. (**b**,**g**) low DNA damage response type with one or two discrete nuclear 53BP1 foci. (**c**,**h**) high DNA damage response type with three or more discrete nuclear 53BP1 foci. (**d**,**i**) diffuse type with heterogeneous and brightly diffuse nuclear staining of 53BP1. E and J: large foci type with discrete nuclear foci measuring ≥1 µm. The scale bars indicate 2 µm.

Following deparaffinization and antigen retrieval by microwave treatment in citrate buffer (pH 6.0), the tissue sections were pre-incubated with 10% normal goat serum. Tissues were then either incubated with a mixture of polyclonal rabbit anti-53BP1 antibody (Bethyl Laboratories, Montgomery, TX) diluted 1:1000 and monoclonal mouse anti-Ki-67 antibody (MIB-1; DakoCytomation, Glostrup, Denmark) diluted 1:100 or with a monoclonal mouse anti-γ histone-2AX antibody (γH2AX; ab26350; Abcam, Tokyo, Japan) diluted 1:1000, a monoclonal mouse anti-p53 antibody (clone DO7; DakoCytomation, Glostrup, Denmark) diluted 1:100, a monoclonal mouse anti-RAD51 antibody (clone 3C10; NEO MARKERS, USA) diluted 1:1000 or a monoclonal mouse anti-DNA-dependent protein kinase, catalytic subunit (DNA-PKcs) antibody (ab1832; Abcam, Tokyo, Japan) diluted 1:100. The samples were then incubated with Alexa Fluor 488-conjugated goat anti-rabbit and/or Alexa Fluor 594 F(abʹ)-conjugated goat anti-mouse antibodies (Molecular Probes Inc., Eugene, OR) as appropriate, and mounted using VECTASHIELD^®^ HardSet Mounting Medium containing DAPI (VECTOR Labs, Burlingame, CA). The stained sections were photographed at 1,000-fold magnification with at least 30 slices per field using the Z-stack function on a High Standard all-in-one fluorescence microscope (BIOREVO BZ-X700; KEYENCE Japan, Osaka, Japan). This enabled the delineation of all 53BP1 foci throughout the nucleus. All signals for 53BP1 expression were measured using the image analysis software provided with the BIOREVO BZ-X700 microscope. 53BP1 immunoreactivity was classified using the following definitions: (1) stable (no staining); (2) low DDR (one or two discrete nuclear foci of <1.0 μm); (3) high DDR (three or more discrete nuclear foci of <1.0 μm); (4) diffuse (intense heterogeneous nuclear staining); or (5) LF (discrete nuclear foci of ≥1.0 μm). Each of these was categorized as either Ki-67 negative or positive, yielding ten distinct categories (Fig. 5)^30,36^. The percentage of cell nuclei containing each type of staining was calculated.

### Multi-colored FISH analysis by UroVysion^TM^

Detection of chromosomes 3, 7, 9p21 and 17 by multi-colored FISH was performed using UroVysion^TM^ (Abbott Japan Co., Ltd., Tokyo, Japan) in accordance with the manufacturer’s instructions; this test employed CEP3 SpectrumRed, CEP7 SpectrumGreen, LSI 9p21 SpectrumGold and CEP17 SpectrumAqua probes. Briefly, following deparaffinization and microwave treatment in citrate buffer (pH 6.0), tissue sections were pre-digested by 0.03% pepsin for 20 min at 37 °C, re-fixed in 4% paraformaldehyde for 5 min at 4 °C, and denatured in 70% formamide for 5 min at 73 °C. Denatured FISH probes were then added and incubated at 37 °C for 16 h. The sections were then washed in 50% formamide and 2× standard saline citrate prior to counterstaining with DAPI (Vysis, Downers Grove, IL, USA). At least 30 slices per field were visualized and photographed using the Z-stack function on the BIOREVO BZ-X700. At least 200 tumor cells were examined in each case. More than two signals of chromosome 3, 7 or 17 per single nucleus were considered to represent a gain, and less than two 9p21 signal per single nucleus was considered to represent a loss. Chromosome aneuploidy was considered to be present in cells with chromosome amplification or loss of >10%.

### Immunohistochemistry

Immunohistochemistry was used to detect p16^ink4a^ expression. Tissue sections were immersed in 0.3% H_2_O_2_ in methanol prior to pre-incubation with 10% normal goat serum. After antigen retrieval, the sections were incubated with purified mouse anti-human p16^ink4a^ antibody (BD Biosciences Pharmingen, San Diego, CA, USA) at a 1:300 dilution for 1 h at room temperature. The slides were subsequently incubated with a biotinylated goat anti-rabbit antibody for 1 h at room temperature before adding avidin-peroxidase and visualizing the diaminobenzidine signal.

### Statistical analysis

The Jonckheere–Terpstra test was used to assess the associations between the type of 53BP1 expression, the tumor grade, and the level of chromosomal instability detected using multi-colored FISH. The cut-off value for differential diagnosis of the histological nuclear grade using the 53BP1 expression ratio was determined by logistic regression. The PHREG procedure in the SAS 8.2 software (SAS Institute, Cary, NC) was utilized for this calculation. All tests were two-tailed, and a p value of <0.05 was accepted as statistically significant.

## Electronic supplementary material

Supplementary Information

## Data Availability

All data generated or analyzed during this study are included in this published article and its Supplementary Information files.
