# Supplementary material for: Expression pattern of p53-binding protein 1 as a new molecular indicator of genomic instability in bladder urothelial carcinoma
Source: Sci Rep. 2018 Oct 19;8:15477. doi: 10.1038/s41598-018-33761-9 (PMC6195620; doi:10.1038/s41598-018-33761-9)
Supplement: Supplementary file 1 — Supplementary Information [file 41598_2018_33761_MOESM1_ESM.docx]

**Supplementary Information**

**Expression pattern of p53-binding protein 1 as a new molecular indicator of genomic instability in bladder urothelial carcinoma**

**Katsuya Matsuda^1^, Tatsuhiko Kawasaki^2^, Yuko Akazawa^3^, Yuhmi Hasegawa^4^, Hisayoshi Kondo^5^, Keiji Suzuki^6^, Masachika Iseki^2^, Masahiro Nakashima^1^***

*^1^Department of Tumor and Diagnostic Pathology, Atomic Bomb Disease Institute, Nagasaki University, Nagasaki, Japan*

*^2^Department of Pathology, Sasebo Kyosai Hospital, Nagasaki, Japan*

*^3^Department of Gastroenterology and Hepatology, Nagasaki University Hospital, Nagasaki, Japan*

*^4^Medical Student Research Program, Nagasaki University School of Medicine, Nagasaki, Japan*

*^5^Biostatistics Section, Division of Scientific Data Registry, Atomic Bomb Disease Institute, Nagasaki University, Nagasaki, Japan*

*^6^Department of Radiation Medical Sciences, Atomic Bomb Disease Institute, Nagasaki University, Nagasaki, Japan*

[*moemoe@nagasaki-u.ac.jp](mailto:*moemoe@nagasaki-u.ac.jp)

This document provides additional figures to support the paper “Expression pattern of p53-binding protein 1 as a new molecular indicator of genomic instability in bladder urothelial carcinoma.”

-
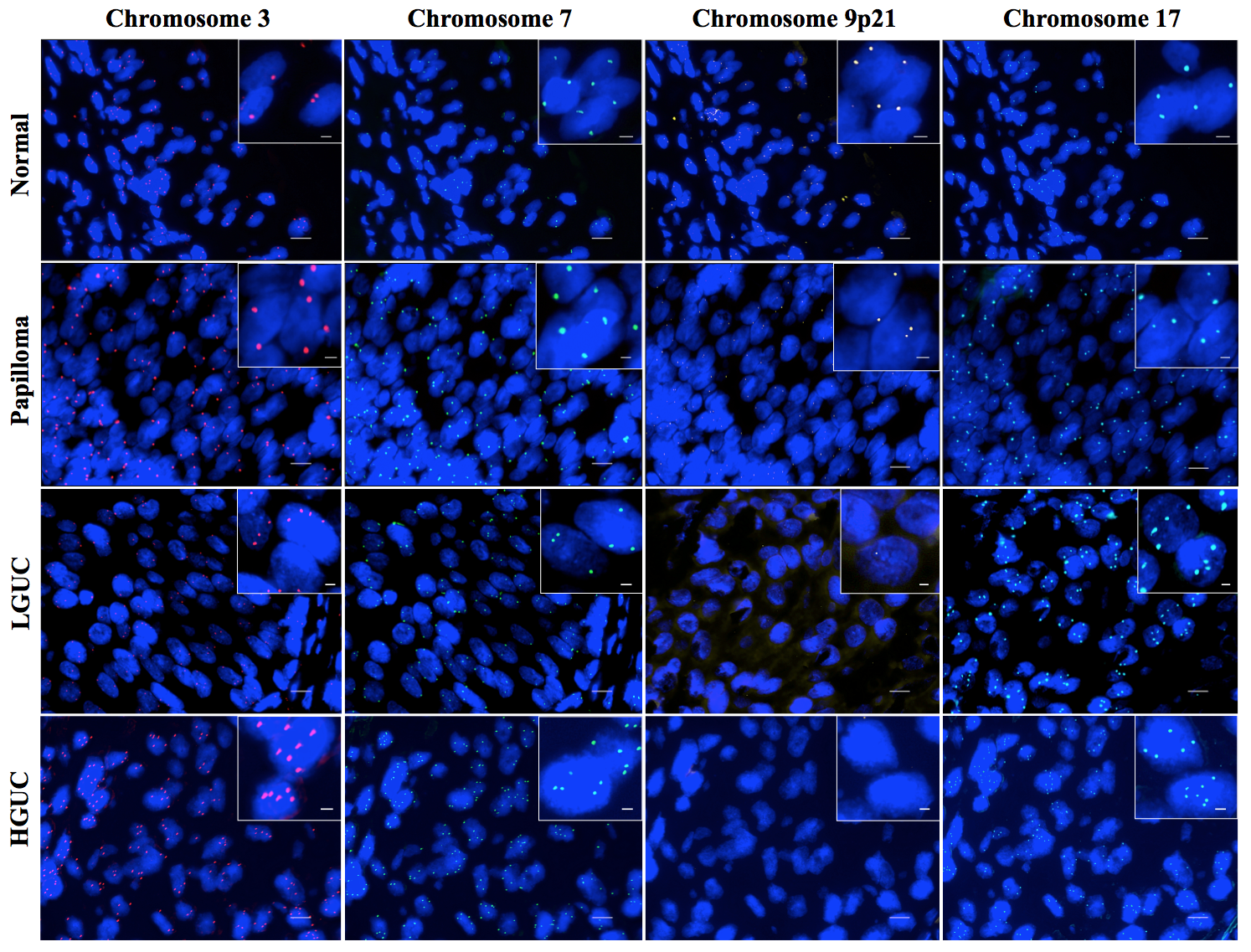

- **Fig. S1.** Representative multi-coloured FISH images of the indicated chromosomes in the indicated urothelial tissues, shown at a lower magnification than in Figure 4. The scale bars indicate 10 µm. The scale bars of inset figures indicate 2 µm. LGUC, low-grade urothelial carcinoma; HGUC, high-grade urothelial carcinoma.
-
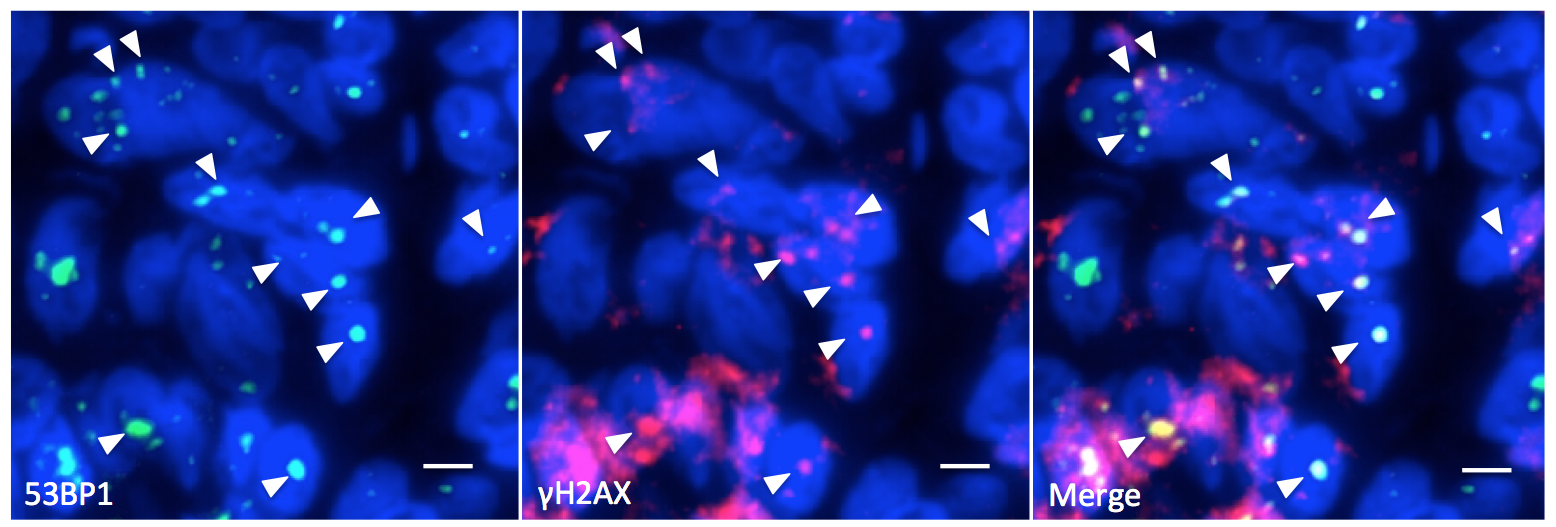
 **Fig. S2.** Double-label immunofluorescence images of the nuclear co-localisation (arrows) of p53-binding protein 1 (53BP1) expression (green) and γH2AX (red) in urothelial papilloma. The scale bars indicate 2 µm.
